# Case report: A 33 years-old alcoholic male with diarrhea and progressive muscle weakness mimicking Guillain–Barré syndrome

**DOI:** 10.3389/fneur.2023.1212497

**Published:** 2023-08-07

**Authors:** Anja M. Rudolph, Sofia Doubrovinskaia, Johannes Knabbe, Corinna Seliger, Thorsten Lenhard

**Affiliations:** Department of Neurology, Heidelberg University Hospital, Heidelberg, Germany

**Keywords:** hyponatremia, Guillain–Barré syndrome, nerve conduction blockade, albuminocytological dissociation, Wernicke encephalopathy

## Abstract

**Background:**

A subacute manifestation of muscle weakness in temporal association with a diarrheal intestinal infection is always suspicious of Guillain–Barré syndrome (GBS). GBS is characterized as an acute inflammatory polyneuroradiculopathy, mediated by cross-reacting autoantibodies and typically triggered by various infections, vaccinations or other causes. Hyponatremia can be associated with GBS and is usually seen in more severe cases. However, the presence of relevant hyponatremia in a case suspicious of GBS can lead to a diagnostic dilemma. We here describe an intriguing and initially misleading case of hyponatremia mimicking GBS, where repeated and thorough electrophysiology was the key to the correct diagnosis.

**Case presentation:**

A 33 years-old man with a history of severe alcohol dependence and schizophrenia developed progressive muscle weakness in the course of a preceding episode of diarrhea. Neurological examination revealed a leg-accentuated tetraplegia with global areflexia. There was also a complex oculomotor dysfunction. Laboratory tests showed hyponatremia of 110 mM. Cerebrospinal-fluid analysis showed a normal cell count and cytological evaluation, protein concentration within the normal range. Electroneurography showed severe proximal nerve conduction block as evidenced by prolonged F-wave latency and distal nerve conduction block as evidenced by prolonged distal motor latencies and reduced motor nerve conduction velocities (NCV) in all peripheral nerves examined. GBS-associated ganglioside autoantibodies were absent. After compensation of hyponatremia alone, muscle weakness improved rapidly and nerve conduction velocity improved similarly. These dynamics are not consistent with GBS and unnecessary immunoglobulin treatment could be avoided.

**Conclusion:**

Suspicion of GBS in the presence of relevant hyponatremia can be misleading as hyponatremia is able to mimic GBS. We demonstrate that repeated and accurate nerve conduction studies together with F-wave diagnostics is helpful to make the correct diagnosis. We discuss the mechanisms of the causes of hyponatremia in GBS and contrast these with the electropyhsiological changes caused by hyponatremia itself. The correct diagnosis will prevent the uncritical use of intravenous immunoglobulins and save unnecessary costs. Also, a possible aggravation of the hyponatremia by immunoglobulin treatment can be averted.

## Introduction

Guillain–Barré syndrome (GBS) is a common cause of acute flaccid paralysis, characterized by symmetric limb weakness and hyporeflexia or areflexia, reaching maximum severity within 4 weeks (1, 2). GBS typically occurs after an infection in which the immune response produces autoantibodies that cross-react with gangliosides on nerve membranes. This autoimmune response leads to a functional blockade of nerve conduction and to subsequent nerve damage. The most common cause of antecedent infection is *Campylobacter jejuni*, but other pathogens such as Epstein-Barr virus, cytomegalovirus, SARS-CoV-2, *Mycoplasma pneumoniae, Haemophilus influenzae* and influenza A virus and many others can also cause GBS (3, 4).

Nerve conduction studies show signs of demyelination, including prolonged distal motor latency (dmL), reduced nerve conduction velocity (NCV), prolonged F-wave latency, increased temporal dispersion and intermediate conduction block (5).

Cerebrospinal-fluid (CSF) analysis reveals a combination of elevated protein levels and normal CSF cell counts (called albuminocytological dissociation), which is considered a hallmark of GBS. In addition, testing for autoantibodies to ganglioside epitopes has been established for the diagnosis of GBS, but has limited positive predictive value because antiganglioside antibodies are also present in other diseases. Exceptions to this rule are anti-GQ1b antibodies, which are present in the serum of at least 90% of patients with Miller–Fisher syndrome as a variant with cranial nerve involvement, and anti-GM1/-GD1a antibodies, which are frequently found in patients with pure motor GBS (1, 6).

In GBS, mild hyponatremia may develop during the course of the disease. Hyponatremia in these patients is more common when mechanical ventilation is required and occurs around day 10 after intubation. Fluid restriction normalizes sodium levels, suggesting a syndrome of inappropriate antidiuresis as the underlying cause. Only a subgroup of GBS patients with severe autonomic dysregulation and extremely high blood pressure have elevated atrial natriuretic protein levels, which may indicate an underlying salt-wasting syndrome (7).

We report a case of severe hyponatremia mimicking features of GBS. However, careful nerve conduction studies revealed an alternative pathogenesis of subacute flaccid tetraparesis despite a history of diarrhea.

## Case description

A 33 years-old man with a history of severe alcohol dependence and schizophrenia was seen by a neurology consultant in the emergency department of a hospital in northern Baden-Württemberg, Germany. The patient reported diarrhea for at least 2 weeks and progressive muscle weakness. He denied fever and associated myalgias or painful paresthesias. He had stopped drinking (6–10 × 0.5 L bottles of beer per day) 2 days before admission. Neurological examination revealed leg-accentuated tetraplegia with global areflexia. Muscle strength was 0/5 for foot and toe flexors and extensors, 2/5 for pelvic girdle muscles and 4/5 for shoulder girdle muscles. Sensory examination revealed a stocking-like hypesthesia of the feet. There was also a complex oculomotor dysfunction with an immured left eye, abduction deficiency of the right eye and a vertical skew deviation. Cognitive assessment revealed mild mental impairment.

Blood analysis showed elevated transaminases, γ-glutamyltransferase, lipase, slightly decreased thiamine level (26 μg/L; *n*: 28–85) and severe hyponatremia (110 mM; *n*: 135–145). Other electrolytes and renal function were within the normal range or borderline (K^+^ 4.4 mM, *n*: 3.3–5.1; Ca^2+^ 1.15 mM, *n*: 1.15–1.35; GFR 57 mL/min, *n*: >60). The CSF on admission showed a normal cell count and cytological assessment, protein concentration within the normal range and an intact blood-CSF barrier. Control lumbar puncture at day 6 after admission showed no changes except an increase in CSF protein (78.2 mg/L, *n*: 15–46). MRI of the entire spine and a CT-scan of the head showed no abnormalities.

**Figure 1:**
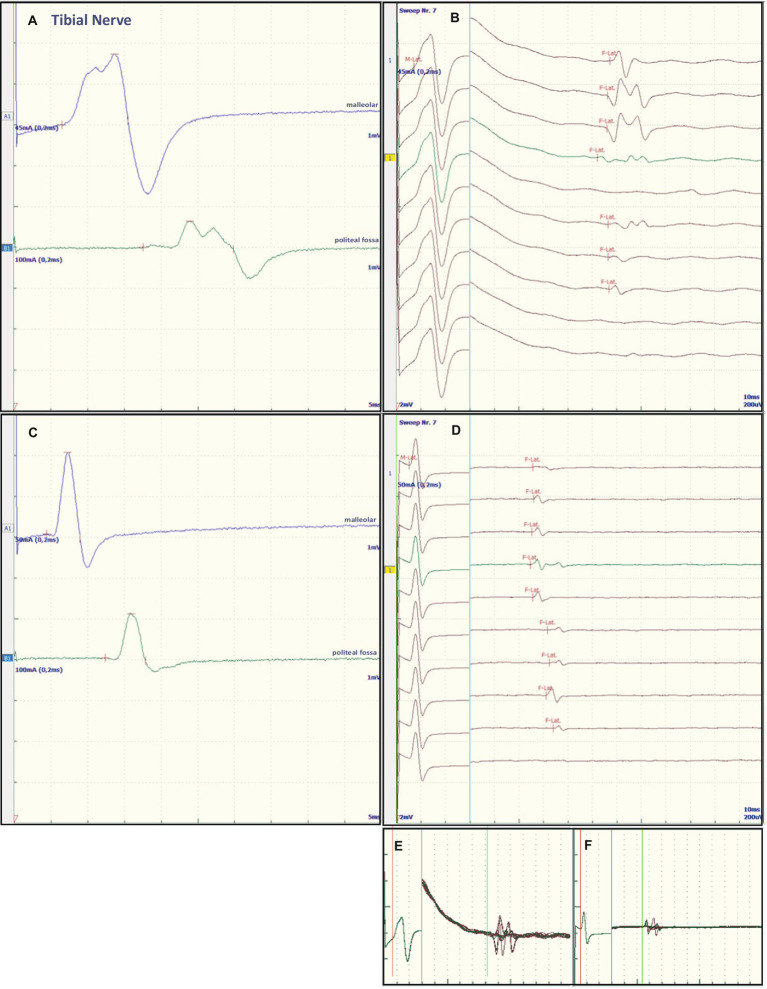
Representative electroneurographic findings for the tibial nerve. **(A,C)** Show nerve conduction velocities with corresponding F-wave latencies **(B,D)**. **(A,B)** Show the findings on admission, **(C,D)** the changes after correction of hyponatremia, 4 days after admission. The small boxes below the F-wave recordings show an overlay of **(B)** F-wave recordings at admission **(E)** and an overlay of **(D)** recordings after sodium corrections **(F)**.

**Figure 2:**
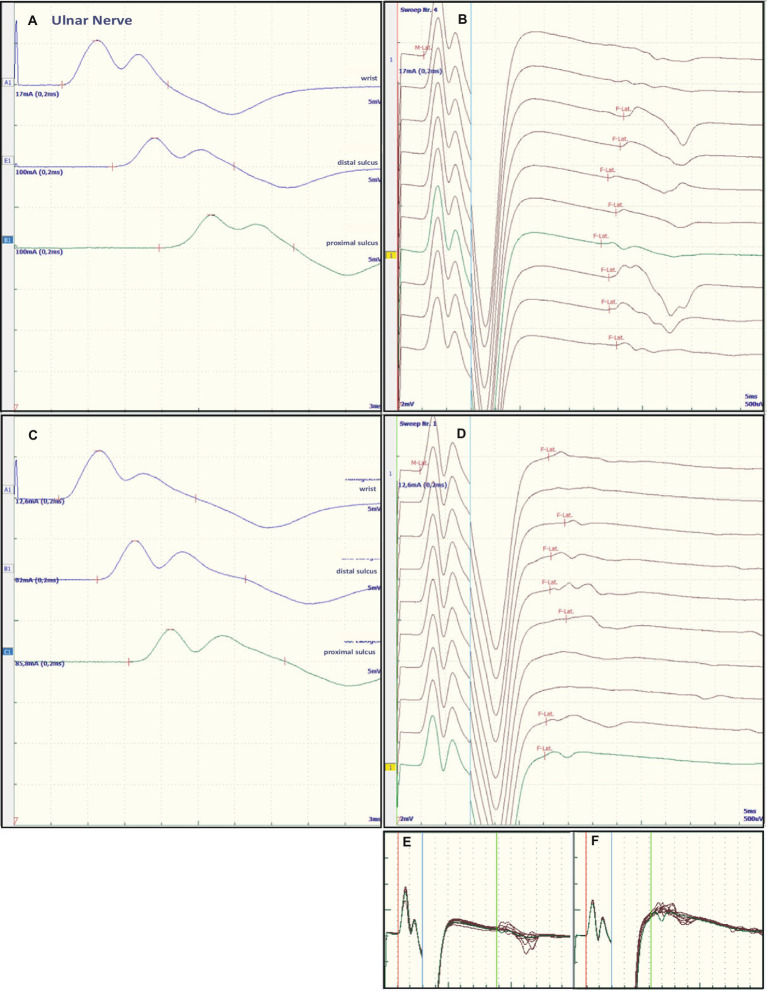
Representative electroneurographic findings for the ulnar nerve. **(A,C)** Show nerve conduction velocities with corresponding F-wave latencies **(B,D)**. **(A,B)** Show the findings on admission, **(C,D)** the changes after correction of hyponatremia, 4 days after admission. The small boxes below the F-wave recordings show an overlay of **(B)** F-wave recordings at admission **(E)** and an overlay of **(D)** recordings after sodium corrections **(F)**.

Nerve conduction studies (NCS) revealed severe proximal nerve conduction block, as evidenced by greatly increased F-wave latency, and distal nerve conduction block, as evidenced by increased dmL and decreased motor NCV in all peripheral nerves studied. Chronodispersion of the tibial nerve compound muscle action potential (CMAP) was also evident. No intermediate conduction block was observed. Figures 1A,B and Figures 2A,B show representative recordings of the ulnar and tibial nerves. Table 1 summarizes the absolute values on admission.

**Table 1:** Summary of electroneurographic findings for ulnar and tibial nerve.

Nerve	Hyponatremia (timepoint)	dmL (msec)	CMAP (mV)	NCV (m/s)	1st F-WL (msec)	MF-W (msec)	F-WCV (m/s)
Ulnar nerve (UN)	Admission^a^	3.9	5.4	39.1	41.6	44.0	5.1
	Compensation^b^	3.6	5.9	44.0	31.6	32.6	7.1
	% changes	−8	+9	+13	−24	−26	+39
Tibial nerve (TN)	Admission^a^	6.6	1.7	33.6	82.2	85.9	2.4
	Compensation^b^	4.4	2.1	44.1	54.7	58.3	4.0
	% changes	−33	+24	+31	−33	−32	+67

dmL, distal motor latency; CMAP, compound muscle action potential; NCV, nerve conduction velocity; F-WL, F-wave latency; MF-W, mean F-wave latency; F-WCV, mean F-wave conduction velocity. Normal values: dmL: UN <4.5 msec, TN <5.4 msec; CMAP: UN >4 mV, TN >5 mV; NCV (age adjusted): UN >42 m/s, TN >40 m/s; 1st F-wave: UN <33 msec, TN <58 msec (leg length adjusted) (8).

a Admission, [Na^+^] 110 mM.

b Compensation, at day 4 after admission, [Na^+^] 136 mM.

Some of the electrophysiological findings are reminiscent of GBS. However, GBS typically has an NCV of less than 30 m/s and often intermediate conduction blocks. Therefore, we concluded that GBS may not be the underlying pathology and that hyponatremia may be the more important underlying pathology. Therefore, we refrained from treatment with intravenous immunoglobulin (IVIG) and compensated the hyponatremia only by slowly increasing the sodium concentration with intravenous sodium infusion. We also replaced the mild thiamine deficiency (200 mg/day intravenously). Within 24 h after admission and the start of sodium replacement [(Na^+^)_0h_ 110 mM; (Na^+^)_24h_ 125 mM; (Na^+^)_48h_ 132 mM; (Na^+^)_72h_ 137 mM], the oculomotor dysfunction and mental abnormalities improved completely. Muscle strength also improved. At the same time, follow-up electroneurography showed an impressive improvement in NCVs and conduction blocks (Figures 1C,D, Figures 2C,D and Table 1).

Further analysis in the course revealed negative results for autoantibodies against gangliosides (IgG + IgM immunoblotting, all negative for GD1a, GD1b, GM1, GM2, GM3, GQ1b, GT1a, GT1b;) and antibodies against *Campylobacter jejuni* (IgM + IgA ELISA: 5.7 and 6.3 U/mL, *n* < 20), making GBS even less likely. The following pathogens were also excluded: *Salmonella-, Yersinia-* and *Shigella species* and *Treponema pallidum, Borrelia burgdorferi*, Hepatitis A, B, C virus and SARS-CoV-2.

The patient’s symptoms improved as described above and he was transferred to a neurological rehabilitation clinic.

## Discussion

This case illustrates severe hyponatremia mimicking GBS. Diarrhea, ascending paresis and mild hypesthesia would strongly remind neurologists of an underlying GBS (1, 2). Although mild sensory deficits may be seen in GBS, more severe sensory deficits, e.g., of the posterior funiculus, are uncommon, whereas muscle pain or painful paresthesias are often associated with GBS. Oculomotor involvement is seen in Miller–Fisher syndrome as a rare variant of GBS (1). Areflexia may also be caused by alcoholic polyneuropathy. Cognitive impairment and oculomotor dysfunction are seen in Wernicke encephalopathy. Other differential diagnoses include myopathies, infectious diseases, malignancies or paraneoplastic neuropathies and disorders of the neuromuscular junction (1). Table 2 summarizes the differential clinical signs and observations in GBS–Miller–Fisher syndrome, Wernicke encephalopathy and hyponatremia with reference to the patient’s particular symptoms on admission.

**Table 2:** Clinical comparison of GBS/Miller–Fisher syndrome, hyponatremia and Wernicke encephalopathy with reference to the patient’s particular symptoms at admission.

Clinical symptom / observation	GBS / Miller–Fisher’s variant	Hyponatremia	Wernicke encephalopathy	Patient
Flaccid paresis	Typical	Impaired motor function, gait instability	Gait instability caused by ataxia, but not flaccid paresis	Present at admission
Ophthalmoplegia	Typical	Not reported	Horizontal gaze nystagmus, INO, abducens nerve paresis	Present at admission
Areflexia	Typical	Reduced tendon reflexes	Not typical^b^	Present at admission
Ataxia	Typical	Rarely reported (9)	Typical	Absent
Cognitive impairment	Not typical	Typical, consciousness disturbance^a^	Typical, consciousness disturbance^a^	Present at admission; not present^a^
Distal hypesthesia	Not typical	Not typical	Not typical	Present at admission
Previous diarrhea	Possible (*C. Jejuni* infection)	Possible as underlying pathology	Not associated	Present at admission
Hyponatremia	In the course possible	intrinsic to disorder	Not associated	Present at admission
Slowed NCV	Typical	Has been described (10, 11)	Not reported^b^	Present at admission
Prolonged F-WL	Typical	Has been described (10, 11)	Not reported^b^	Present at admission
Prolonged dmL	Typical	Has been described (11)	Not reported^b^	Present at admission
Thiamine deficiency	Not associated	Not associated	intrinsic to disorder	Borderline
Albuminocytological dissociation	Typical in the course, often not present at the time of admission	Not associated	Not associated	Present in the course

INO, internuclear ophthalomoplegia; NCV, nerve conduction velocity; F-WL, F-wave latency; dmL, distal motor latency.

a Refers to a symptom mentioned within a cell of the same line.

b Axonal neuropathy has been described in beriberi (19, 20), but exact data on NCV, F-WL, dmL are not available.

Sodium disturbances, an important laboratory finding in our patient, are known to be associated with GBS (12). A syndrome of inappropriate antidiuresis is discussed as the underlying pathology in most cases, and a salt-wasting syndrome has been reported in isolated cases (7, 13, 14). However, to our knowledge, hyponatremia mimicking GBS in clinical signs and some electroneurographic patterns has not been published (15), yet.

In most cases, hyponatremia in GBS is mild and is associated with a severe course of the disease and mechanical ventilation or with IVIG treatment (7, 13, 16). In our patient, however, the hyponatremia was severe (110 mM) and was already present on admission. It is most likely due to massive beer consumption in the sense of dilutional hyponatremia. As the symptoms in our patient resemble Miller–Fisher syndrome, which is strongly associated with *C. jejuni* or other pathogens and GQ1b and GT1a ganglioside autoantibodies, the absence of these antibodies in our patient did not support this diagnosis (17); nor did the absence of albuminocytological dissociation at admission. Other causative agents of GBS were also excluded. Finally, convincing evidence was provided by the rapid improvement in oculomotor dysfunction and muscle weakness with sodium replacement, which occurred much faster than would be expected in GBS with IVIG treatment.

The NCS showed changes that are common in GBS. However, some features raised differential diagnostic considerations. There were severe proximal conduction blocks, but only a slightly reduced NCV, which was still faster than 30 m/s, and no intermediate conduction blocks. Therefore, after sodium correction, there was also a rapid improvement in follow-up NCS, as shown for proximal nerve conduction blocks and NCV (Figures 1C,D and Figures 2C,D). These electroneurographic changes usually improve much more slowly (if at all) in GBS. They are associated with severe disease progression (e.g., mechanical ventilation) and delayed improvement (2).

Most neurological manifestations of hyponatremia are neurocognitive and mental status changes (confusion, disorientation, seizures, syncope, coma) and not necessarily neuromuscular, with the exception of unsteady gait (18). The most likely cause of unsteadiness leading to falls is decreased reaction latency, secondary to decreased NCV and F-wave latencies (10), as were also present in our patient at admission (see Table 1, Figures 1A,B and Figures 2A,B). Previous publications have shown reversibility of NCV and F-wave latencies in hyponatremia with correction of sodium levels (10, 11). However, our patient had a pre-existing relevant axonal polyneuropathy, which was still evident in reduced CMAP even after compensation for hyponatremia, and this finding was particularly accentuated in the tibial nerve (see Table 1 and Figures 1A,B). Here, the NCV slowing and the F-Wave and dmL extension were particularly pronounced [compare changes in percentage (%) in tibial nerve versus ulnar nerve, Table 1]. This suggests that the pre-damaged nerve is particularly susceptible to hyponatremia, which clinically results more in a manifesting flaccid paresis than just gait instability. This is supported by the fact that the effect was particularly evident in the tibial nerve, where the CMAP remained pathologically reduced, whereas the ulnar nerve had a normal CMAP at baseline but also showed an increase in CMAP with sodium correction (Table 1). These results are consistent with the measurements made by Vandergheynst et al. (10), who discuss in their paper the effect of pre-existing polyneuropathy on patients’ symptoms and electrophysiological changes in hyponatremia.

The contribution of thiamine deficiency to the severity of symptoms, particularly ocular motor dysfunction, needs to be discussed. Since thiamine levels were only slightly decreased, we believe that thiamine deficiency may have had some effect, but the main pathology was due to hyponatremia. A contribution of osmotic demyelination to the observed ophthalmoplegia could be ruled out by evaluation of the clinical course. A CT-scan of the head at day 6 showed normal brainstem structures including pons. In chronic thiamine deficiency (beriberi) in alcoholic patients or pregnant women rapid improvement of neurological symptoms including muscle weakness has been reported after replacement of thiamine (19, 20). One study reports patients predominantly presenting with axonal polyneuropathy (19). The other reports women exclusively presenting with axonal polyneuropathy (20). However, neither study mentions quantitative values of electrophysiological measurements or of thiamine concentrations. Rather, as discussed above, the pre-existing alcoholic polyneuropathy in our patient may have exacerbated the effects of hyponatremia on the peripheral nerves rather than a relevant thiamine effect, showing reversible proximal and distal nerve blockades as so-called “demyelinating features” (Figures 1, 2 and Table 1). The rapid improvement of these “demyelinating features” with sodium replacement shows that the changes are reversible and not due to substantial damage to the nerve fibres, further supporting the hyponatremia hypothesis.

Our key message is that if severe hyponatremia is present already on admission, the diagnosis of GBS should be questioned. An attempt to treat the sodium balance alone may be indicated, as IVIG treatment can further exacerbate the hyponatremia (2, 21). In addition, other IVIG-associated side effects may be avoided and costs saved. Furthermore, the case is very instructive because it illustrates the nature of the multifactorial causality (hyponatremia, pre-existing alcoholic neuropathy, thiamine deficiency) of the entire clinical picture. Developing a multifactorial mindset is critical to formulating optimal differential diagnoses, a medical skill rarely taught in textbooks.

## Data availability statement

The original contributions presented in the study are included in the article/supplementary material, further inquiries can be directed to the corresponding author.

## Ethics statement

Ethical review and approval was not required for the study on human participants in accordance with the local legislation and institutional requirements. The patients/participants provided their written informed consent to participate in this study.

## Author contributions

TL conducted and evaluated the electrophysiological studies and drafted the manuscript. AR, SD, and JK conducted the clinical and laboratory examinations and proofread the manuscript. CS performed a second evaluation of the electrophysiological examinations and proofread the manuscript. All authors contributed to the article and approved the submitted version.

## Conflict of interest

The authors declare that the research was conducted in the absence of any commercial or financial relationships that could be construed as a potential conflict of interest.

## Publisher’s note

All claims expressed in this article are solely those of the authors and do not necessarily represent those of their affiliated organizations, or those of the publisher, the editors and the reviewers. Any product that may be evaluated in this article, or claim that may be made by its manufacturer, is not guaranteed or endorsed by the publisher.
